# From trash to treasure: tumor draining lymph nodes as a multi-omics goldmine in cancer therapy

**DOI:** 10.3389/fonc.2025.1636942

**Published:** 2025-08-19

**Authors:** Yina Li, Zihan Chen, Zhikun Guo, Jiangnan Yu, Jianan Lu, Lei Wang, Qian Zhou

**Affiliations:** International Cancer Center, Shenzhen University Medical School, Shenzhen, Guangdong, China

**Keywords:** TDLN, immunotherapy, radiotherapy, chemotherapy, LNMPR, multi-omics technology

## Abstract

Tumor draining lymph nodes (TDLNs), as secondary lymphoid organs, are pivotal in initiating and regulating adaptive immune responses. Historically, TDLNs were recognized primarily as metastasis gateways in cancer, promoting radical dissection to prevent recurrence. However, emerging preclinical studies reveals their critical role in orchestrating systemic anti-tumor immune responses during cancer therapy, highlighting the dilemma of balancing lymph nodes (LNs) preservation with metastasis control. This review traces the evolving understanding of TDLN biology in oncology, from the era of radical LN dissection to multi-omics-driven insights, and synthesizes their dual roles as immune hubs and metastatic niches across first-line clinical therapies (e.g., immunotherapy, radiotherapy, chemotherapy, targeted therapy, etc.). We further propose the concept of “Lymph Node Multi-modal Protective Research (LNMPR)”, emphasizing the prospective value of integrating multi-omics technologies, including spatial transcriptomics, single-cell profiling, and imaging, to decode LN immune dynamics and optimize therapeutic responses. By bridging mechanistic insights with clinical strategies, LN-centric immune modulation may open up a new path for precise tumor treatment.

## 1 Introduction

Despite advancements in clinical cancer therapies, including immunotherapy, radiotherapy, chemotherapy, and targeted therapy, improving the overall survival of cancer patients remains an urgent challenge, with tumor metastasis serve as the major causes of treatment failure and patient death. Tumor draining lymph nodes (TDLNs), interconnected via lymphatic vessels, acting as gateways for tumor dissemination, occupy a paradox roles as immune hubs and metastatic stations (1). They filter antigens through lymphatic drainage while eliminating infections and antigenic substances. At the same time, enabling immune cells trafficking and adaptive immune response initiation. The above basic functions shape the unique role of TDLNs in antitumor immunity. On the one hand, due to the open structure of lymphatic vessels, tumor cells are easily drained and colonized into lymph nodes to form LNs metastasis, and s reached through lymphatic vessels are recognized as TDLNs. On the other hand, immune cells in the TDLNs are activated by the draining tumor antigens, triggering anti-tumor immunity, which will have an important impact on tumor development and anti-tumor immunity. This duality positions TDLNs at the epicenter of cancer progression and therapy—harboring metastatic seeds yet simultaneously priming anti-tumor T cells (2). In this review, TDLNs encompass both non-metastatic and metastatic LNs, as both play distinct yet critical roles in tumor progression, immunity, and therapeutic responses.

The classical metastasis paradigm posits that LNs involvement precedes distant spread, justifying radical LN dissection. For decades, procedures like radical mastectomy prioritized en bloc LNs removal, driven by the perception of LNs as “metastatic garbage dumps”. However, genomic analysis in colorectal and breast cancers reveal that distant metastases often arise independently of LN clones, challenging the necessity of routine dissection (3, 4). Long-term clinical data further question this approach: LN dissection in breast cancer and melanoma fails to improve survival while increasing postoperative complications, such as lymphedema and the immune suppression caused by impaired antigen presenting cells (APC) function (5, 6). For instance, axillary dissection reduces peripheral CD8^+^ T cells by 37%, impairing immune surveillance (7). These findings highlight a paradigm shift—LN metastasis may primarily serve as a prognostic biomarker rather than a causal driver of progression (8).

**Figure 1:**
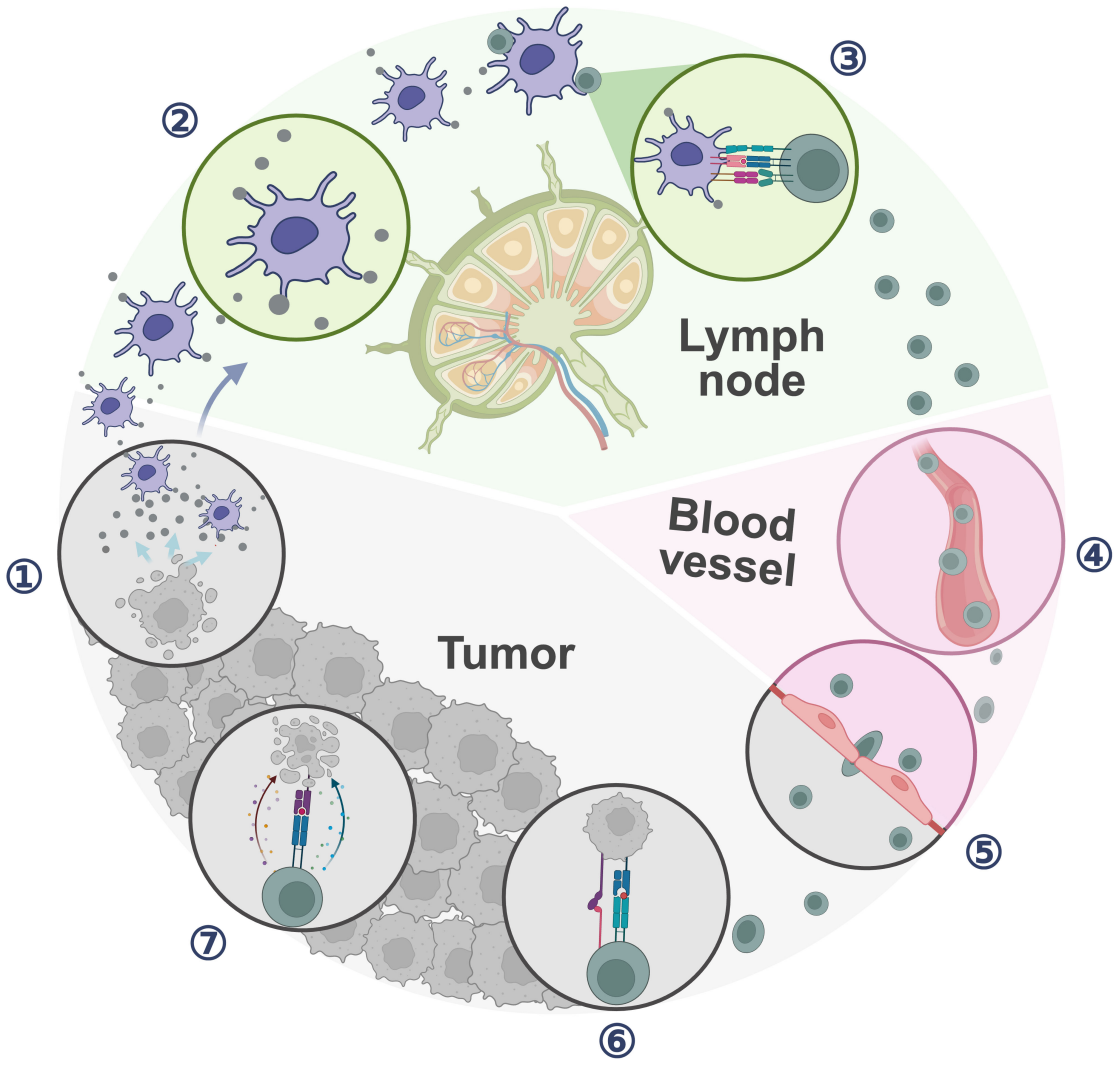
Cancer immune cycle. ① Cancer cell releases tumor antigens; ② APCs capture and process tumor antigens; ③ APCs sensitize and activate T cells; ④ T cells traffic through blood vessel; ⑤ Antitumor T cells infiltrate the tumor; ⑥ T cells recognize cancer cells; ⑦ T cells kill cancer cells. (Created with bioRender.com).

Cancer Immunity Cycle (CIC) highlights the role of TDLNs as orchestrators of systemic anti-tumor immunity rather than merely metastasis sites. This perspective necessitates a reevaluation of the importance of TDLNs in immune cell monitoring and priming for immune responses (**Figure 1**). During clinical anti-tumor therapy, dead tumor cells released antigens are captured by dendritic cells (DCs), which migrate to TDLNs to prime naive T cells. Subsequently, naive T cells proliferate and differentiate into effector T cells and memory T cells. Those activated effector T cells then exit the LNs and infiltrate the tumor, specifically recognizing and killing tumor cells while releasing additional antigens, while memory T cells can survive for a long time *in vivo*, and can rapidly proliferate and differentiate into effector T cells to play a role in immune response when the same antigen is encountered again, facilitating the CIC positive feedback loop (9). TDLNs thus act as “training center” for effector T cells and hubs for innate-adaptive crosstalk, that collectively influence treatment outcomes (10). The sentinel lymph node (SLN) refers to the first LN receiving lymphatic drainage directly from the primary tumor (11).

Clinically, in breast cancer and melanoma surgeries, tracer agents are used to locate and guide the excision of the SLN to assess nodal metastasis, guiding decisions on whether axillary lymph node dissection (ALND) should be performed (12). The sentinel lymph node biopsy (SLNB) exemplifies the shift from indiscriminate removal to precision preservation of LN function. Clinically, SLNB significantly reduced the incidence of postoperative lymphedema from 35–40% to 5–7% while maintaining equivalent tumor control, underscoring the therapeutic value of LN preservation (13). Moreover, these clinically obtained lymph node specimens have also become invaluable materials for evaluating TDLN immune dynamics and decoding the immunological roles of TDLNs.

Traditional techniques like flow cytometry, immunohistochemistry, and immunofluorescence provides limited insights into LN complexity due to limited detection throughput or lack of cellular interaction information. The multi-omics revolution, encompassing single-cell RNA sequencing, spatial transcriptomics, and proteomics unveiled LNs as not only a “relay station” for tumor metastasis but also an “decision-making center” and “training camp” for systemic immune regulation. The cellular interactions and molecular mechanisms within TDLN influence anti-tumor treatment. For instances, single-cell sequencing analysis of head and neck cancer patients’ tissues identify precursor exhausted T cells (T_pex_) with stem cell characteristics positioned near DCs in uninvolved LNs, which could differentiate into intermediate exhausted T cells (T_ex-int_) and terminal exhausted cells (T_ex_) post anti-PD-L1 immunotherapy and infiltrate tumor. In contrast, in metastatic LNs, these cells are surrounded by an immunosuppressive microenvironment, impairing their activation and differentiation capabilities (14). Similarly, spatial proteomic analysis of breast cancer primary tumors and paired LN metastases reveals survival-associated cellular phenotypes (e.g., p53^high^ or GATA3^high^) outperforming traditional clinical classification criteria (such as TNM staging or molecular subtypes) (15).

Currently, the role of LNs in oncology is undergoing a cognitive revolution: from being regarded as “metastatic trash” that need to be completely eliminated in the past, LNs have been gradually transformed into a treasure of the body’s anti-tumor immunity, which is not only reflected in the optimization of surgical strategies (e.g., the popularization of SLN biopsy), but also driven by the panoramic view of the immune microenvironment of TDLNs revealed by multi-omics techniques. Future therapeutic strategies need to move from “removal” to “preserve” and explore how to fully mount the antitumoral immune potential of TDLNs when optimizing cancer elimination strategies. Here, we systematically summarize the immunological changes and underlying mechanisms of TDLNs across different therapeutic modalities from a multi-omics perspective, centering on the pivotal role of LNs in cancer therapy. We further propose the core concept of Lymph Node Multi-modal Protective Research (LNMPR), which aims to elucidate the cellular composition, microenvironment, and intercellular interactions within TDLNs through integrative multi-modal analyses. In conclusion, we hope this review may offer a more comprehensive foundation for clinical precision therapy, while providing potential insights and translational implications. By bridging mechanistic discoveries with clinical translation, TDLNs are expected to serve as critical allies for enhancing anti-tumor immunity.

## 2 The role of TDLNs in tumor occurrence and development

Increasing evidence supports the view that cancer is not confined to its primary site but manifests as a systemic disease (16). TDLNs, located at the intersection of peripheral tissues and systemic immunity, are among the earliest immune structures to be reprogrammed by tumor-derived signals, even before metastasis occurs (17). These signals initiate a cascade of structural and immunological changes that precede detectable metastasis. Rather than merely filtering lymphatic fluid, TDLNs actively participate in shaping disease progression by undergoing premetastatic remodeling—including stromal reorganization, lymphangiogenesis, and immunoregulatory cell recruitment—that prepares the TDLN niche for subsequent tumor cell colonization. Dissecting how these changes unfold across distinct tumor types is essential for understanding the mechanisms that convert TDLNs from immunological sentinels into metastasis-permissive environments.

### 2.1 Changes in the TDLN microenvironment

Tumor-derived factors can dynamically reshape the immune architecture of TDLNs well before metastatic tumor cells arrive, initiating early immunological remodeling that favors immune evasion and future dissemination. As tumors progress, interactions between the tumor microenvironment (TME) and the broader immune system lead to immunosuppressive remodeling in TDLNs. These alterations facilitate future metastatic dissemination and compromise anti-tumor immune responses. In breast cancer, single-cell and spatial multi-omics profiling of metastatic lymph nodes (LNMTs) revealed profound suppression of T cell activation, cytotoxicity, and proliferative capacity compared to primary tumors (PT). Within LNMTs, CD4+ CXCL13+ T cells are more prone to differentiate into an exhausted state, and LAMP3^+^ DCs exhibit a reduced ability to prime T cells relative to their counterparts in the PT (18). In oral squamous cell carcinoma (OSCC), SPP1^+^ tumor-associated macrophages (TAMs) activate fibroblasts via SPP1-CD44 signaling and induce T cell exhaustion by promoting the ubiquitination and degradation of CD226 on T cells through CD155-CD226 signaling. These processes collectively remodel the TDLNs microenvironment to support tumor cell colonization and proliferation (19). Moreover, tumor cells metabolically adapt to the fatty acid-rich TDLN microenvironment. A YAP-driven fatty acid oxidation (FAO) program supports their energy needs and survival. Multi-omics and functional studies further revealed that YAP-FAO metabolic adaptation and bile acid-VDR signaling promote TDLN metastasis and may be targetable by agents such as everolimus (20).

The TDLN microenvironment serves as a permissive niche that fosters the seeding and outgrowth of metastatic cancer cells (1). The microenvironment of TDLNs with different tumors types exhibits specific changes. For example, before metastasis, breast cancer-draining LNs first enlarge and then shrink, accompanied by changes in T-cell activation levels, leading to an immunosuppressive microenvironment (21). In prostate cancer LN metastases (22), the proportion of CD8+ T cells is decreased, whereas the proportions of regulatory T cells (Tregs) are increased, leading to an immunosuppressive state. In lung adenocarcinoma LN metastases (23), which are classified into different subtypes based on morphological features, the immune microenvironment includes collagenous and necrotic types. Interestingly, TDLN stromal cells may retain immunostimulatory capacity by secreting CCL21, thereby promoting T cell clustering and activation (24). Furthermore, in melanoma, tumor-induced stromal reprogramming drives TDLN transformation. Specifically, fibroblastic reticular cells (FRCs) within TDLNs proliferate and undergo structural remodeling and transcriptional modifications in response to tumor-derived factors. These changes include the downregulation of key chemokines like CCL21 and IL-7, leading to altered immune cell composition and localization within the TDLNs, and form an immunosuppressive microenvironment to promote tumor metastasis (25).

### 2.2 The metastatic pathways of tumor cells

Molecular alterations in the TDLN microenvironment not only reflect tumor progression but also actively drive metastatic dissemination. Tumor cells hijack specific chemokine axes and adhesion pathways to home to TDLNs and breach lymphatic barriers. A key axis is CCL21-CCR7, where lymphatic endothelial cells secrete CCL21 to attract CCR7^+^ tumor cells. This interaction activates the PI3K/AKT and MAPK/ERK1/2 pathways, leading to cytoskeletal remodeling and enhanced motility. Meanwhile, in melanoma models, CXCL12-CXCR4 signaling from perilymphatic stromal cells boosts invasiveness via Rho GTPase activation (e.g., RhoA, Rac1) (26). In addition, tumor cells also degrade the lymphatic basement membrane through proteases such as matrix metalloproteinases (MMPs) and cathepsins, facilitating transendothelial migration. Notably, silencing MMP-2 in breast cancer significantly reduced LN invasion (27). Adhesion molecules further stabilize interactions with lymphatic endothelial cells, for instance, integrin α4β1 binds to Vascular Cell Adhesion Molecule-1 (VCAM-1), while L1CAM-mediated Src kinase activation facilitates transendothelial migration. Inhibiting these interactions (e.g., blocking integrin α4) effectively limits TDLN colonization (28).

## 3 The role of TDLNs in immunotherapy

While tumor cells rely on the lymphatic system to facilitate metastasis, particularly TDLNs, simultaneously serve as key sites for initiating and regulating antitumor immune responses. This dual role positions TDLNs as critical hubs where tumor progression and immune surveillance intersect. Gaining a deeper understanding of the cellular and molecular dynamics within TDLNs is therefore essential for uncovering mechanisms of immune escape and improving the efficacy of immunotherapeutic strategies (**Table 1**).

**Table 1:** Modalities of immunotherapy, LN changes and multi-omics research methods in cancers.

Cancer type	Species	Treatment	Drug name	Multi-omics approaches	LN changes
Melanoma (24)	Human	anti-CTLA4/anti-PD1	Ipilimumab/Nivolumab/Pembrolizumab	scRNA-seq/DSP	↑TLS, ↑activates B and T cell responses
Melanoma (71)	Human	anti-CTLA4/anti-PD1	Nivolumab/Ipilimumab	scRNA-seq	TDLNs: ↑CD5^+^DCs
Melanoma (72)	Human	anti-CTLA4/anti-PD1/anti-CTLA4+anti-PD1	\	scRNA-seq/ATAC-seq/TCR-seq/WES	\
Melanoma (73)	Mice	anti-CD4	GK1.5	scRNA-seq/TCR-seq/Bulk RNA-seq	TDLNs: ↑tumor-specific Trm
Melanoma and RCC (74)	Human	anti-PD1/anti-CTLA4/anti-PD1+anti-CTLA4	nivolumab/nivolumab+ipilimumab/nivolumab+ bevacizumab	scRNA-seq/Bulk mRNA- seq/CyTOF/DSP/	\
Breast cancer (68)	Mice	anti-PD1/anti-CTLA4	Anti-mouse PD1 (clone RMP1-14)/Anti-mouse CTLA4 (clone 9D9)	scRNA-seq/Bulk mRNA-seq	SLNs: ↑T follicular helper, ↑ B cells, ↑IgG and ↑T cell
NSCLC (75)	Mice	anti-PD1/anti-IL4	BioXcell, clone 10F.9G2/BioXcell, clone 11B11	scRNA-seq/CITE-seq	TDLNs: ↑mregDCs
HNSCC (14)	Human	anti-PDL1	atezolizumab	scRNA-seq/TCR-seq/CITE-seq/CyTOF	uiLN: ↓T_pex_ and ↑T_ex-int_

Symbols indicate trends in LN changes reported in cited studies. ↑: increase, ↓: decrease. These changes are based on qualitative descriptions rather than precise numerical values.

DSP, Digital Spatial Profiling; TLS, tertiary lymphoid structures; ATAC-seq, Assay for Transposase-Accessible Chromatin with high-throughput Sequencing; TCR-seq, T Cell Receptor sequencing; WES, T Cell Receptor sequencing; Trm, resident memory T cells; RCC, Renal Cell Carcinoma; CyTOF, Cytometry by Time Of Flight; NSCLC, Non-Small Cell Lung Cancer; CITE-seq, Cellular Indexing of Transcriptomes and Epitopes by Sequencing; mregDCs, Mature Regulatory Dendritic Cells; HNSCC, Head and Neck Squamous Cell Carcinoma; uiLN, uninvolved lymph node; T_pex_, Progenitor exhausted T cells; T_ex-int_, Intermediate exhausted T cells.

### 3.1 TDLNs: battle between immunosurveillance and immune tolerance

TDLNs are centrally regulated for antitumor immunity, serving as both a “training ground” for T cell activation and a “sanctuary” for immune evasion. In TDLNs, naive T cell activation requires the coordination of two key signals (29). The first signal comes from antigen-presenting cells (APCs) presenting peptide antigens via MHC I/II and binding to T cell receptors (TCRs) for antigen-specific recognition (30). This interaction ensures antigen-specific recognition by T cells. However, TCR signaling alone is not sufficient for full activation and additional regulatory mechanisms are required. The second signal arises from the interaction of co-stimulatory and co-inhibitory molecules, which together determine the fate of the T cell (31). A typical co-stimulatory signal is the binding of CD28 on the surface of T cells to CD80/CD86 on the surface of APCs, which enhances TCR-mediated activation signals and promotes T cell proliferation and initiation of effector functions (32). In contrast, co-inhibitory signaling inhibits T cell over-activation through negative regulation. For example, upon T cell activation, the inhibitory receptor CTLA-4 is induced to be expressed, which binds CD80/CD86 with higher affinity than CD28 but does not transmit activation signals, thereby blocking CD28-mediated costimulation and negatively regulating T cell responses. These co-inhibitory molecules, collectively known as immune checkpoints, play a crucial role in maintaining immune homeostasis and preventing autoimmunity.

At the same time, LN induces immune tolerance through multiple mechanisms: it is considered to be a central site for coordinating tolerogenic immune responses (33). In TME, TDLNs usually establish a state of tolerance to tumor-specific T cells by generating peripheral inducible regulatory T cells (iTreg), which inhibit their activation and expansion. In addition, stromal components such as FRC and lymphatic endothelial cells (LEC) in LNs can promote apoptosis or incapacitation of autoreactive T cells by presenting peripheral tissue antigens and activating the programmed cell death protein 1 (PD-1)/programmed death-ligand 1 (PD-L1) pathway. While these inhibitory mechanisms help maintain immune homeostasis and prevent autoimmunity, they may also provide a breeding ground for immune escape by tumor cells. Therefore, as a dual hub of immune response, TDLN needs to maintain a fine balance between stimulating anti-tumor immunity and maintaining immunosuppression.

### 3.2 The immunoregulatory role of immune checkpoint pathways in TDLNs

Immune checkpoints are protective molecules within the human immune system that function as “brakes”, preventing excessive activation of T cells that could otherwise lead to inflammatory damage. Typical immune checkpoint molecules include PD-1, PD-L1 and CTLA-4. PD-1/PD-L1 is one of the key immune checkpoint pathways. PD-1 is an inhibitory receptor primarily expressed on activated T cells, B cells, certain natural killer cells, activated monocytes, DCs, and certain macrophages (34–37). PD-L1 is one of the ligands for PD-1, expressed on tumor cells, stromal cells, and immune cells (such as lymphocytes and myeloid cells). Under normal physiological conditions, the interaction between PD-L1 and PD-1 transmits inhibitory signals that prevent excessive immune responses (30, 38). In tumor immune evasion, the PD-1/PD-L1 axis inhibits immune activation in TDLNs through the following mechanisms: (1) suppressing the expression of co-stimulatory molecules on DCs (e.g., CD80/CD86, CD40); (2) reducing the activity of the ZAP-70 and PI3K/Akt pathways in the TCR signaling cascade; (3) interfering with the activation and proliferation of precursor exhausted T cells (T_pex_ cells) and blocking their differentiation into intermediate exhausted T cells (T_ex-int_ cells); (4) disrupting spatial interactions between cDC1 and T_pex_ via the CXCL9/10-CXCR3 axis; (5) maintaining the immunosuppressive state of Tregs.

Immune Checkpoint Inhibitors (ICIs) immunotherapy represents a revolutionary advancement in oncology (39). The typical immune checkpoint inhibitors primarily include monoclonal antibodies targeting PD-1, PD-L1, and CTLA-4. Their mechanism of action primarily involves targeting inhibitory receptors on immune cells, releasing the “brakes” on T cells, thereby restoring their antitumor cytotoxic function and re-establishing the immune activation network within TDLNs. These antibodies have now been approved for clinical treatment of various solid tumors, such as melanoma (40, 41), non-small cell lung cancer (42–44), renal cell carcinoma (45, 46), and triple-negative breast cancer (47, 48).

### 3.3 Myeloid APCs in TDLNs

Myeloid APCs, which include several subpopulations such as DCs and macrophages, are specialized cells that acquire, process, and present antigens to naive T cells to induce antigen-specific immune responses (49). The role of APCs in naive T cell activation is crucial. Cytometry by time-of-flight and single-cell RNA sequencing of peripheral blood from patients with hepatocellular carcinoma (HCC) demonstrated that immunotherapy efficacy is closely interwined with different APCs and memory T cells (T_mem_) (50). Considering that the TDLN serves as an important site for the intersection of APCs and T cells, it would be more promising to explore the effects of APCs-T interactions on ICI in the TDLN.

In the functionally diverse antigen-presenting cell system, DCs play a central role in anti-tumor immunity during ICI therapy by migrating from the tumor and initiate the anti-tumoral response. On the one hand, DCs can capture antigens in the TME and migrate to the TDLN to activate naive T cells to initiate an immune response; on the other hand, tumor-derived antigens can also be directly drained to the TDLN and cross-presented by resident DCs, which in turn activate effector CD8^+^ T cells. Van Pul et al. further revealed the critical role of DCs in breast cancer immunotherapy. They found that in the SLN of breast cancer, the resident conventional DC subpopulation (LNR-cDC) showed suppressed activation (e.g., decreased CD86 expression) before the tumor had metastasized, and this suppression preceded the exhaustion of T-cell effector function, suggesting that the LNR-cDC may be a key target for breaking through immunosuppression and restoring anti-tumor immunity (7). Preclinical studies have demonstrated that ICI efficacy relies on synergistic effects between DCs (especially the cDC1 subset) and T cells. After PD-1 blockade, activated CD8^+^ T cells secrete IFN-γ to activate cDC1, which induces them to produce IL-12. IL-12 can further enhance CD8^+^ cytotoxic T cell function, thereby promoting the recovery of T cell dysfunction in anti-PD-(L)1 therapy (51). Furthermore, cDC1 not only efficiently uptakes and delivers tumor antigens to the TDLN to activate CD8^+^ T cells, but also secretes chemokines locally in the tumor to recruit T cells and maintains their survival and function through cytokines. For instance, Jan P. Bottcher’s team identified the intra-tumor cDC1-CD8^+^ T-cell population as a key component of protective anti-tumor immunity based on RNA-seq and deep learning approaches, and identified a unique population of immune-stimulatory CCR7^neg^ cDC1s that produce CXCL9 to promote CD8^+^ T cell population recruitment and drive T by cross-presentation of tumor antigen cell differentiation and expansion (52).

Despite the dominance of cDC1 in antitumor immunity, the potential of cDC2 in antitumor immunity should not be overlooked. In the cDC1-deficient mouse model, transcriptional profiling revealed an activation state of CD11b^+^ cDCs expressing an activation state of CD11b^+^ cDCs—interferon stimulated gene (ISG) signature (ISG^+^ DCs). Unlike cDC1, which activates CD8^+^ T cells through cross-presentation, ISG^+^ DC is able to acquire and present an intact tumor-derived peptide-MHC I complex and has the ability to activate CD8^+^ T cells in a manner comparable to cDC1, as well as promote protective anti-tumor immunity in the absence of cDC1. In addition, the ISG^+^ DC gene signature can be detected in human tumors. Importantly, in poorly immunogenic tumors lacking cDC1, ISG^+^ DC can be induced by the addition of exogenous IFN-β to drive anti-tumor CD8^+^ T cell responses (53). Moreover, in head and neck squamous cell carcinoma (HNSCC) patients treated with anti-PD-L1, T_pex_ and DCs were more closely spatially co-localized in uninvolved lymph nodes (uiLNs) (14), which further emphasizes the critical role of DC-T cell interactions on ICI efficacy. In melanoma patients, CD141^+^ DCs, as the most migratory DC subpopulation, deliver tumor antigens to the TDLN via a CCR7-dependent pathway, and are a key cell type for initiating CD8^+^ T cell responses (49). All of the above studies support that enhancement of DCs function can be an important strategy to improve the efficacy of ICI.

In addition to DCs, macrophages would also act as APCs. Asano et al. demonstrated that CD169^+^ subcapsular sinus (SCS) macrophages are able to phagocytose dead tumor cells transported through the lymphatic fluid in the TDLN and cross-present tumor antigens to CD8^+^ T cells to activate anti-tumor immune responses (54). However, in the mouse model, the absence of CD169^+^ macrophages lead to impaired activation of tumor-specific CD8^+^ T cells, which cannot-boost anti-tumor immunity. Meanwhile, during tumor progression, tumor-derived extracellular vesicles (tEVs) propagate through the lymphatic system and preferentially bind to SCS CD169^+^ macrophages in TDLNs. The CD169^+^ macrophage layer physically prevents the propagation of tEVs, but the barrier is disrupted by tumor progression and certain therapeutic agents. When the SCS macrophage barrier is disrupted, tEVs can enter the TDLN cortex and interact with B cells to promote tumor-promoting humoral immunity. Thus, CD169^+^ macrophages have important therapeutic potential for their tumor-suppressive role in curtailing tEV propagation and limiting tumor-promoting immunity. Notably, the immunoregulatory role of PD-L1^+^ macrophages may not be uniformly suppressive. In human breast tumors, PD-L1-expressing TAMs were more mature, spatially proximate to CD8^+^ T cells, and capable of promoting their proliferation and cytotoxicity. These PD-L1^+^ TAMs were associated with better relapse-free survival, suggesting that PD-L1 expression on myeloid cells may, under certain conditions, reflect an immunostimulatory phenotype rather than immune suppression (55). This functional dichotomy illustrates the nuanced roles of macrophage subsets in modulating anti-tumor immunity, suggesting that selectively targeting distinct myeloid populations may hold therapeutic potential.

### 3.4 CD8^+^ T cells

ICIs blocked the PD-1/PD-L1 pathway can relieve the suppression of T cells, enhance their ability to receive co-stimulatory signals, and promote the activation, proliferation, and cytotoxic (56). More importantly, ICIs not only restore T cell function but also promote the maintenance or reversion of some CD8^+^ T cells to a stem cell-like precursor state TCF-1^+^ T_pex_, endowing them with higher proliferative and lineage plasticity potential within TDLNs. These activated T_pex_ cells can then migrate into the TME, where they continue to exert cytotoxic functions after differentiating into T_ex-int_ cells, thus driving the closure of the CIC (14, 57).

In recent years, the development of multi-omics technologies has significantly advanced our understanding of the immune mechanisms of T_pex_ cells. Rahim et al. Rahim et al. conducted integrated single-cell transcriptome, spatial transcriptome, and TCR lineage tracing analysis of human head and neck squamous cell carcinoma (HNSCC) patients cohort and found that CD8^+^ T_pex_ cells were mainly distributed in cDC1-enriched regions in uninvolved regional lymph nodes (uiLNs).These T_pex_ cells are the first to be activated following PD-L1 blockade and represent a critical effector population. Upon receiving signals from DCs, these T_pex_ cells are activated and differentiate into the T_ex-int_ lineage, while entering the peripheral blood and migrating to the TME to re-engage tumor antigens, contributing to the exhausted T cell (T_ex_) pool population and sustaining antitumor immune functions (14, 58). Further research indicates that compared to metastatic LNs, T_pex_ cells in uiLNs exhibit significant activation and lineage expansion following PD-L1 inhibition, whereas metastatic LNs display marked immunosuppression, impeding the functional remodeling of T_pex_ cells. Additionally, changes in T_pex_ levels are highly consistent with the dynamic evolution of the T_ex_ population in the peripheral circulation and TME, suggesting that anti-PD-L1 treatment activates the differentiation and proliferation of T_pex_ cells within TDLNs and promotes their release into the bloodstream, driving the CIC in a positive direction. It can be inferred that T_pex_ cells in TDLNs are highly responsive to ICIs, and T_pex_ cells in TDLNs that have not been invaded by tumors have the potential to predict ICI responses.

In addition to T_pex_ cells, a study used single-cell multi-omics methods to report the existence of tumor antigen-specific memory CD8^+^ T cells in the TDLNs of a mouse tumor model. One subset of these cells, characterized by the immune phenotype PD-1^+^ TCF-1^+^ TOX^–^, exhibits features of classical T_mem_ cells. These cells were named Tumor Draining Lymph Node-derived Tumor-Specific Memory T cells (TDLN-TTSM) (59). The study found that PD-L1 inhibitors promote the substantial expansion of TDLN-TTSM cells, leading to the accumulation of T_pex_ and T_ex_ cells in the TME, thereby demonstrating that TDLN-TTSM cells are the true responders to ICIs. The research also showed that removing TDLNs during the neoadjuvant treatment phase, such as before surgery, significantly inhibits the expansion of TTSM cells, almost completely abolishing the efficacy of PD-L1 blockade. This indicates that TDLNs play an irreplaceable role as an activation hub in early treatment responses.

In summary, CD8^+^ T cell subsets within TDLNs, particularly T_pex_ and TDLN-TTSM, serve not only as critical hubs for initial antigen response and effector cell output in the CIC but also as a “reservoir of response” in ICIs treatment. Multi-omics approaches, by providing high-dimensional insights into their spatial localization, lineage tracing, and immune interaction networks, offer a robust mechanistic foundation and clinical translational potential for individualized treatment decisions based on TDLN status, such as preserving TDLNs and predicting ICIs efficacy.

### 3.5 CD4^+^ T cells

In the CIC, CD4^+^ T cells not only function as effector cells exerting direct antitumor effects but also regulate other immune cells to construct a systemic immune response network. Tumor antigens, after being captured by DCs, migrate to TDLNs and are presented to naive CD4^+^ T cells via MHC II proteins, initiating their activation and differentiation programs. Within TDLNs, CD4^+^ T cells differentiate into various subsets, predominantly Th1 cells, which secrete cytokines such as IFN-γ, IL-2, and TNF. These cytokines not only enhance CD8^+^ T cell cytotoxicity but also stimulate DC activation and directly inhibit tumor cell growth (60). Activated CD4^+^ T cells further enhance antigen presentation and activate more T_pex_ or CD8^+^ T cells through CD40L-CD40 interactions with DCs, forming a positive feedback loop that drives the CIC toward deeper responses (60, 61). Compared to CD8^+^ T cells, CD4^+^ T cells exhibit greater functional diversity and phenotypic plasticity, bridging local immune responses in TDLNs with systemic immunity (57, 62). Among CD4^+^ T cell subsets, T follicular helper (Tfh) cells in TDLNs interact with B cells to deliver survival signals, promoting their proliferation, differentiation, and affinity maturation, thereby enabling B cells to produce high-affinity antibodies against tumor antigens. This enhances humoral immune responses and contributes to anti-tumor immunity (21, 63).

Tregs, an immunosuppressive subset of CD4^+^ T cells, are prevalent in TDLNs, particularly in advanced cancer patients, where their numbers and expression of immune checkpoint molecules are significantly elevated. Tregs are critical mediators of immune suppression in tumors, and correlates with poor patient prognoses. Tregs can suppress DCs activation by highly expressing inhibitory receptors such as CTLA-4, PD-1, and TIGIT. For instance, CTLA-4 can impair DCs function by either competitively binding to CD80/CD86 or depleting them from the DCs surface via trans-endocytosis, thereby limiting the DCs’ ability to provide costimulatory signals to T cells. These mechanisms contribute to the suppression of CD8^+^ T cell and Th1 cell effector functions and play a crucial role in tumor immune evasion (64, 65). ICI therapy, particularly anti-CTLA-4 antibodies, disrupts this suppression by blocking the interaction between CTLA-4 and B7 molecules, thereby relieving the inhibition of co-stimulatory pathways between DCs and T cells and amplifying subsequent immune responses (66). Moreover, the proportion and activity of Tregs in TDLNs have been shown to be critical predictors of ICI efficacy. Expansion of Tregs in TDLNs is often associated with resistance to ICIs, while their reduction or functional inhibition correlates with better treatment responses (65, 67). Therefore, monitoring Tregs in TDLNs can aid in predicting ICI efficacy and guiding personalized treatment strategies.

In the context of immunotherapy, the ratio, spatial distribution, and functional state of Th1 and Tregs within TDLNs can be precisely characterized using single-cell transcriptomics or spatial transcriptomics, emerging as key indicators for assessing immunotherapy responses and prognosis.

### 3.6 The role of B cells in immunotherapy

In addition to T cells, the main component of the adaptive immune system consists of B cells. B cells capture and process tumor antigens via their surface B cell receptors (BCR) in the TDLNs, and present antigens to CD4^+^ T cells through MHC II proteins, thereby activating T cell-mediated immune responses. In specific immune microenvironments, B cells can form germinal centers within the TME or TDLNs, which is the core of adaptive humoral immunity. Additionally, Hollern et al. found that in a breast cancer model, the activation of B cells in TDLNs is closely related to the production of antigen-specific antibodies and significantly enhances the therapeutic effect of PD-1 blockade, a process that depends on the activation, differentiation, and establishment of humoral immune responses of B cells (68). Compared with T cells, research on B cell immune checkpoints in tumors is still in its infancy (69). Some studies have identified TIM-1 as a key checkpoint for B cell activation. TIM-1 affects type I interferon responsiveness in B cells, restricting B cell activation, antigen presentation, and co-stimulation, thus highlighting TIM-1 as a potential target. By targeting this checkpoint, B cell responses can be unleashed to promote anti-tumor immunity. Within TDLNs, blocking TIM-1 relieves immune suppression of B cells, enhancing their antigen-presenting and co-stimulatory functions, which in turn indirectly activates T cell anti-tumor responses. The combined use of TIM-1 and PD-1 inhibitors can simultaneously relieve immune suppression of both B cells and T cells, resulting in a stronger anti-tumor effect (70). Additionally, in a breast cancer mouse model, the primary tumor is capable of inducing a significant accumulation of B cells in the TDLN, which begins as early as 1 week after tumor inoculation and precedes the detection of tumor cells in the TDLN. This further reveals that B cells in the TDLN promote the migration and LN metastasis of tumor cells by producing pathogenic IgG antibodies that activate the CXCR4/SDF1α axis within tumor cells (28). This indicates that B cells in the TDLN are not only involved in the formation of the pre-metastatic niche but also directly facilitate tumor metastasis.

## 4 The role of TDLNs in radiotherapy

Radiotherapy not only targets tumor cells but also exerts profound effects on TDLNs, influencing both local tumor control and systemic immune responses. This section outlines its direct cytotoxic effects within TDLNs, its role in shaping the immune microenvironment, and its clinical synergy with LN dissection.

### 4.1 Direct effects of radiotherapy on tumor cells within TDLNs

Radiotherapy is not only a key modality for directly eliminating tumor cells, but is also increasingly recognized as a crucial tool for modulating the immune microenvironment of TDLNs. Radiotherapy directly targets tumor cells in the TDLNs using high-energy radiation (such as X-rays or gamma rays), causing DNA damage and cell death through various mechanisms, such as apoptosis, necrosis, and autophagy (70). Radiotherapy induces DNA double-strand breaks (DSBs) in tumor cells, activating the DNA damage response (DDR) pathway within the tumor cells (24, 28). The key molecules that activate DSBs are Ataxia Telangiectasia Mutated (ATM) and Ataxia Telangiectasia and Rad3-related (ATR), which further regulate downstream signaling pathways (71–73). ATM/ATR phosphorylate molecules such as p53 and CHK1/CHK2, leading to cell cycle arrest (e.g., G1/S or G2/M phase arrest) or apoptosis (74, 75). Conventional fractionated radiotherapy (e.g., 2 Gy per fraction) effectively eliminates tumor cells but concurrently damages T cells and DCs within TDLNs, leading to lymphopenia and immunosuppression which would be benefit for tumor recurrence. Therefore, optimizing dose fractionation strategies is essential to keep balance tumor eradication while preservation of immune function (76).

### 4.2 Radiotherapy remodels the immune microenvironment of TDLNs

In addition to directly killing tumor cells and functional immune cells, radiotherapy can indirectly induce potent anti-tumor immune responses by modulating the immune microenvironment of TDLNs (76). For instance, radiotherapy triggers immunogenic cell death (ICD), leading to the release of damage-associated molecular patterns (DAMPs) such as HMGB1 and ATP, which promote DC maturation and antigen presentation, thereby reshaping the immune landscape within TDLNs. Radiotherapy also upregulates the expression of CXCL9 and CXCL10 TDLNs, facilitating the recruitment of effector T cells and natural killer (NK) cells, and enhancing immune recognition and clearance of tumor cells (77). Moreover, tumor-derived double-stranded DNA (dsDNA) can be released into DCs via exosomes or cytoplasmic transfer, activating the cGAS/STING/IFN-I signaling pathway and promoting DCs migration to TDLNs, which further amplifies CD8^+^ T cell responses (78, 79). The effective radiotherapy would boost the CIC and immune response.

In recent years, there has been increasing focus on neoadjuvant radiotherapy strategies that irradiate only the primary tumor while sparing TDLNs to preserve their immune function. Khalifa et al. conducted a retrospective study in patients with localized cN0 NSCLC, implementing neoadjuvant concurrent chemoradiotherapy (CRT) directed solely at the primary tumor while preserving LNs (80). The results revealed that in non-irradiated, non-involved TDLNs, more than 1,000 differentially expressed genes were identified compared to patients who did not receive neoadjuvant CRT. These genes were enriched in pathways related to anti-tumor immunity, inflammatory responses, hypoxia, angiogenesis, epithelial–mesenchymal transition, and extracellular matrix remodeling. Furthermore, the gene expression profiles in preserved TDLNs were closely associated with better pathological responses, suggesting that sparing TDLNs during radiotherapy may help maintain crucial immune niches and thereby promote systemic anti-tumor immune responses. These findings support the potential benefits of node-sparing CRT strategies in NSCLC treatment and may positively impact patients’ therapeutic responses and prognoses.

### 4.3 Clinical synergistic effects of radiotherapy combined with LN dissection

In HNSCC, the combination of radiotherapy and LN dissection has been shown to significantly improve patient survival. This therapeutic strategy effectively eliminates locoregional lymph node metastases and reduces the risk of tumor recurrence (81, 82). The immune response induced by radiotherapy, when combined with LN dissection, can further enhance systemic anti-tumor immunity. Radiotherapy not only facilitates antigen release via direct tumor cell killing but also reshapes the TDLNs microenvironment, such as by activating DCs and to promote T cell-mediated immune responses. Simultaneously, LN dissection removes immunosuppressive metastatic niches, synergistically enhancing treatment efficacy (83). Radiotherapy plays a dual role in LN-associated tumor therapy: on the one hand, it directly induces tumor cell apoptosis. On the other hand, it activates immune pathways that remodel the functional state of TDLNs. Although early radiotherapy may impair TDLN follicles and T cell populations, the stromal and vascular architecture is often preserved, providing a structural basis for subsequent immune restoration. Emerging studies also suggest that hypofractionated radiotherapy regimens, such as FLASH radiotherapy, which uses an ultra-high dose rate for ultra-fast radiotherapy, may minimize nonspecific damage to immune cells and better preserve the immunological potential of TDLNs (8).

In summary, radiotherapy serves not only as a critical modality for local tumor control but also as a potent enhancer of anti-tumor immunity through activation of immune circuits within TDLNs. This immunomodulatory role becomes particularly prominent when radiotherapy is combined with LN dissection strategies. Looking forward, integrating multi-omics approaches (such as spatial transcriptomics and TCR tracking) to dynamically characterize immune alterations within TDLNs holds great promise for advancing “precision radiotherapy” toward an “immunologically integrated precision radiotherapy” paradigm.

## 5 The role of TDLNs in chemotherapy

Chemotherapy has long been recognized for its direct cytotoxicity against tumor cells, yet growing evidence reveals that its therapeutic efficacy also hinges on the host immune system—particularly the functional integrity of TDLNs. As central hubs for antigen presentation and T cell priming, TDLNs participate in the CIC by processing ICD-derived signals and coordinating systemic immune responses. However, chemotherapy exerts a double-edged effect on the immune landscape of TDLNs: while it can deplete lymphocytes and disrupt antigen presentation, it may also activate DCs and effector T cells via damage-associated molecular patterns (DAMPs) and type I interferon pathways. Using common regimens such as anthracycline-taxane combinations as representative models, this section explores the dual roles of TDLNs during chemotherapy, highlighting their contribution to immune activation and their vulnerability to immunosuppressive remodeling.

### 5.1 LN-dependent immune activation during chemotherapy

Conventional chemotherapy exerts antitumor effects by interfering with tumor cell metabolism, inhibiting mitotic progression, inducing DNA damage, and triggering apoptotic pathways (84, 85). For instance, anthracyclines mediate DNA double-strand breaks through intercalation into DNA and inhibition of topoisomerase II (86), while taxanes disrupt mitotic processes by stabilizing microtubule structures (87). Chemotherapy can also induce ICD, promoting the release of DAMPs that activate APCs. These APCs migrate to TDLNs, where they prime tumor-specific T cells, initiating antitumor immune responses critical for the CIC (9, 88, 89). Impaired LN function or inefficient antigen presentation can disrupt this process, compromising post-chemotherapy immunity (90).

Neoadjuvant chemotherapy (NAC) currently represents the standard therapeutic approach for high-risk early-stage, locally advanced, or inoperable breast cancer. Administered preoperatively, NAC aims to reduce tumor burden and assess therapeutic sensitivity. Multi-omics analyses of breast cancer have revealed dynamic genomic and transcriptomic changes associated with neoadjuvant chemotherapy (NAC) response, identifying biomarkers such as CDKAL1 P409L, ADGRA2, and ADRB3 linked to chemoresistance and prognosis (91). Although these studies focused on tumors and peripheral blood without investigating lymph nodes, this highlights a research gap and the need to further explore immune dynamics within TDLNs during chemotherapy. Consequently, understanding and preserving key immune populations in TDLNs may be essential for enhancing chemotherapy efficacy.

### 5.2 Chemotherapy-induced remodeling of TDLNs immunity: a double-edged sword

Chemotherapy exerts paradoxical effects on TDLN-resident immune cells. On the one hand, certain agents (e.g., cisplatin, alkylating agents) induce broad lymphocyte depletion, impairing antigen presentation and T-cell functionality (92). A study showed that 88% of breast cancer patients had key immune cell levels remaining below 70% of the normal range even nine months after chemotherapy (93). On the other hand, specific chemotherapeutics such as anthracyclines can promote ICD, releasing DAMPs like HMGB1 and ATP (90). These DAMPs can be captured by DCs in the TDLNs and activate DC maturation and migration through the cGAS-STING-IFN-I pathway, thereby inducing the establishment of CD8^+^ T cell effector responses (94, 95). Moreover, chemotherapy may upregulate immune checkpoint molecules such as PD-L1, enabling tumor immune evasion and contributing to the establishment of an immunosuppressive microenvironment. Conversely, it can also selectively deplete immunosuppressive Tregs and enhance the infiltration of effector T cells into TDLNs, thereby improving local immune responses (96, 97). Clinical studies have shown that combining chemotherapy with PD-1/PD-L1 inhibitors can significantly enhance the activation of immune cells within TDLNs. For example, paclitaxel combined with a PD-1 inhibitor has been shown to boost T cell anti-tumor activity (98). These findings highlight the dualistic nature of chemotherapy’s impact on the TDLN immune landscape, which can both impair immune surveillance and potentiate antitumor immunity through immune activation. Optimizing the synergy between chemotherapy and immunotherapy remains a critical goal in cancer treatment research.

The widely used “anthracycline plus taxane” regimen in breast cancer exemplifies this complexity. Multiple studies have shown that this combination substantially reshapes the TDLNs immune microenvironment. Anthracyclines robustly induce ICD, promote antigen cross-presentation, and activate the DC-T cell axis within sentinel TDLNs. Taxanes, in contrast, may modulate chemokine expression and lymphocyte trafficking, thereby affecting the dynamic redistribution of T cells between TDLNs and the TME (86, 87). This regimen has been proven to significantly reduce LN metastasis (99), with studies reporting an approximately 30% increase in LN metastasis control rates in breast cancer patients (100). However, despite its immunostimulatory benefits, this treatment strategy may also exert immunosuppressive effects, potentially inhibiting T cell activity and reducing immune cell numbers within TDLNs, thus weakening local immune surveillance (101). Therefore, balancing the dynamic interplay between immune activation and immune exhaustion during chemotherapy may represent a key strategy for improving therapeutic outcomes.

## 6 The role of TDLNs in targeted therapy

Targeted therapy, which precisely intervenes in the molecular pathways driving tumor development and progression, has become an important treatment strategy following chemotherapy. Common targets include HER2, EGFR, and VEGF, which play key roles in tumor metastasis, especially in LN metastasis. As an early site of tumor metastasis, the microenvironment within TDLNs not only affects drug distribution but also contributes to the heterogeneity of therapeutic responses.

### 6.1 The mechanism of targeted drugs in inhibiting tumor cells in TDLNs

Take breast cancer as an example, HER2 overexpression is one of the most common oncogenic driver events. HER2-targeted therapeutic drugs (such as trastuzumab) significantly inhibit tumor cell proliferation and metastasis by blocking HER2 dimerization and inhibiting signaling pathways such as PI3K/AKT and MAPK (102). In addition, HER2-targeted antibodies can also mediate ADCC, eliminating HER2-positive tumor cells by activating NK cells or macrophages. This process occurs in TDLN metastases with high HER2 expression, and the response to targeted drugs is closely related to local immune activity (103). EGFR-targeted therapy is often used for EGFR-mutated tumors such as NSCLC, with representative drugs including gefitinib and erlotinib. These drugs inhibit cell growth and metastasis by competitively binding to the EGFR tyrosine kinase domain and blocking downstream RAS/MAPK and PI3K/AKT signaling pathways (104). Studies have shown that EGFR expression levels in TDLNs, along with the immune activity of the local microenvironment, may influence drug efficacy. This may partly explain the primary or acquired resistance to EGFR inhibitors observed in certain patients (28).

### 6.2 The regulation of the immune microenvironment of TDLNs by targeted therapy

Targeted therapy not only acts directly on tumor cells, but also indirectly regulates anti-tumor immunity by influencing immune cells and the stromal environment within the TDLNs. The VEGF pathway is widely regarded as the key to regulating tumor angiogenesis. Anti-vegf drugs (such as bevarizumab) can promote vascular normalization, improve microcirculation in TDLNs, and enhance drug permeability (20). Meanwhile, VEGF inhibition can also reduce the infiltration of immunosuppressive cells (such as Tregs and Myeloid-derived suppressor cells) in TDLNs, promote the maturation of antigen-presenting cells such as cDC1, and enhance the immune response mediated by T cells (104).

### 6.3 The clinical significance of HER2-targeted therapy in LN metastatic breast cancer

In HER2-positive breast cancer, the LN metastasis status is an important indicator affecting treatment decisions. Multiple clinical studies have shown that HER2-targeted therapy significantly reduces the positive rate of LN and improves overall survival (OS) and disease-free survival (DFS) (103). Antibody-drug conjugates such as T-DM1 (ADCs) overcome the limitations of the therapeutic effect on some TDLNs immunosuppressive microenvironments by precisely delivering cytotoxic drugs to HER2-positive TDLNs metastases (105). In addition, combined treatment strategies such as trastuzumab combined with pertuzumab can enhance the immune response in TMEs and TDLNs, improve the therapeutic effect and delay the occurrence of drug resistance (102).

## 7 Conclusion and future perspectives

This review discusses the intricate interactions between TDLNs and tumor immunity in clinical first-line treatments revealed by multimodal spatial multi-omics technologies, and clarifies that TDLNs are important sites that affect tumor metastasis and the response to anti-tumor treatments. These studies highlight how lymph nodes paradoxically promote tumor cell colonization while also triggering systemic immune responses. During immunotherapy, radiotherapy, chemotherapy and targeted therapy, the microenvironment and cellular composition of TDLNs undergo dynamic changes, and these changes profoundly affect the outcome and survival of patients. For example, the success of ICI treatment is closely related to the activation status of CD8^+^ T cells in TDLNs, especially the key role of stem cell-like T_pex_ cells in immunotherapy (106, 107). Similarly, CD4^+^ T cells, Tregs and B cells, etc. are also involved in the complex immune regulatory network in TDLNs (25).

**Figure 2:**
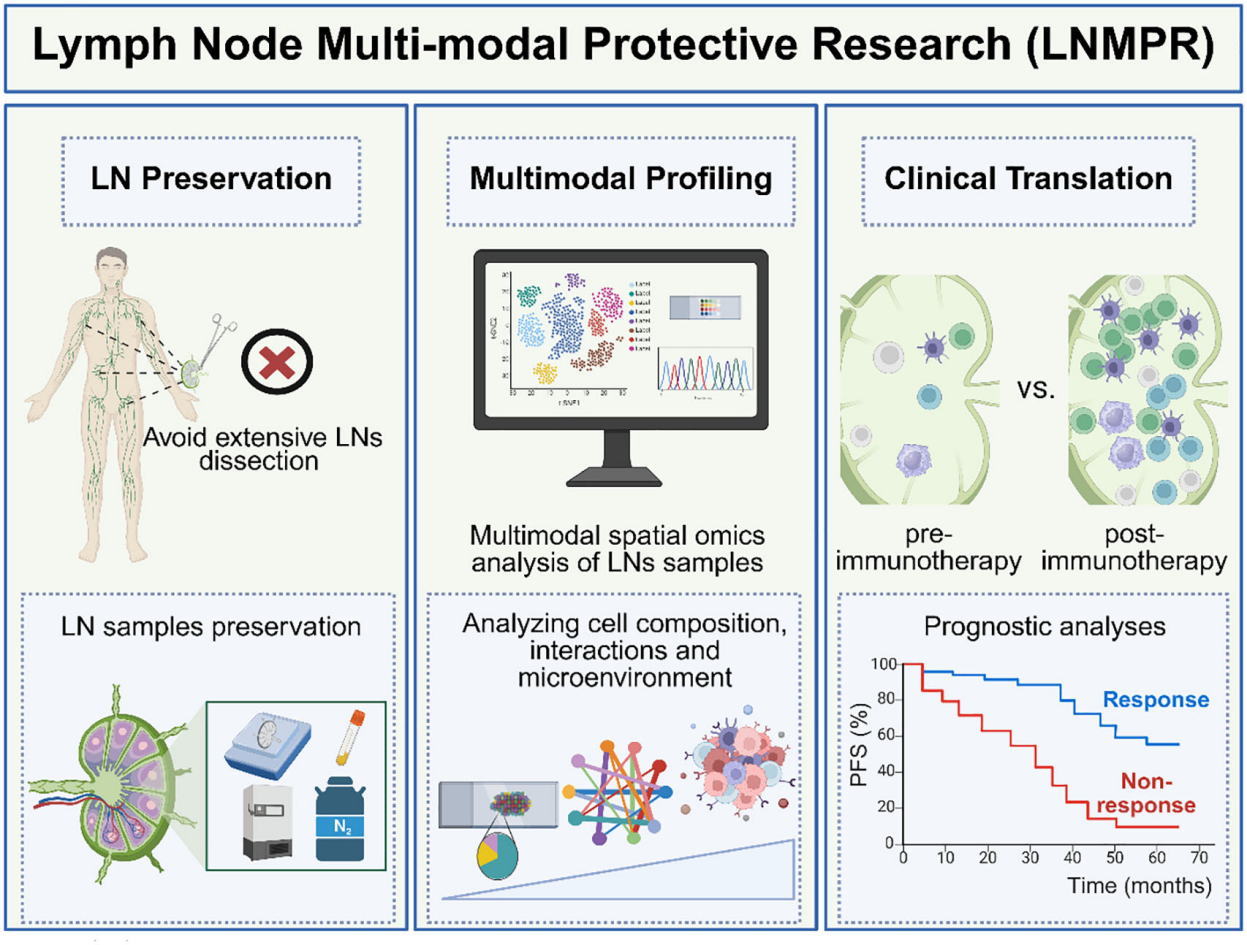
The framework of Multimodal Protective Study of Lymph Nodes (LNMPR). (Left panel) LN Preservation: Selective dissection strategies seek to minimize surgical damage to immune architecture. The LN samples obtained during surgery should be stored properly. (Medium panel) Multimodal Profiling: We can use multimodal spatial omics to analyze LN samples and further analyze cell composition, interactions, and microenvironment within the LN. (Right panel) Clinical Translation: The characteristics of immune cells in the LNs of the non-response group and the response group were compared as biomarkers related to treatment response. The progression-free survival (PFS) time was compared between the response group and the non-response group. (Created with bioRender.com).

To harness this duality, future strategies must prioritize precision interventions targeting TDLN microenvironments. Therefore, we proposed the Multimodal Protective Study of Lymph Nodes (LNMPR) framework offers a transformative approach, integrating LN preservation, multimodal analysis, and clinical translation. LNMPR emphasizes three pillars (**Figure 2**).

### 7.1 LN preservation

Avoiding extensive surgical dissection to maintain immune competence, as exemplified by a set of “six rules” guidelines for selective LN resection proposed by Prof. Chen (108). Concurrently, advanced cryopreservation and biobanking protocols ensure high-quality LN samples for downstream analyses. In addition to surgical strategies and tissue preservation, emerging minimally invasive approaches such as liquid biopsy may further support the assessment of LN status and immune function. Emerging liquid biopsy methods—including ctDNA and exosome analysis—show promise for minimally invasive monitoring of systemic and LN-specific immune dynamics. For example, lymphatic drainage fluid cfDNA has demonstrated greater sensitivity than plasma in detecting tumor recurrence risk (109). While evidence linking liquid biopsy to TDLN remodeling is limited, integrating these tools into the LNMPR framework could enhance patient stratification and precision therapy.

### 7.2 Multimodal profiling

Combining fresh and FFPE samples enables comprehensive dissection of LN biology. Fresh tissues facilitate single-cell transcriptomic and proteomic analyses, while FFPE specimens support spatial omics and histopathological mapping. This integration resolves cellular heterogeneity (e.g., Cell functional differences such as T/B cell differentiation dynamics, stromal-immune crosstalk, genomic-level or epigenetic-level heterogeneity) and microenvironmental cues (chemokines, cytokines) while preserving spatial context. Cutting-edge tools like Spatial-Mux-seq, which simultaneously captures histone modifications, chromatin accessibility, transcriptomes, and proteins spatially at cellular level (110), are poised to decode the epigenetic and functional states of immune activated or immunosuppressive niches (e.g., antigen-presenting cell enriched regions and Treg-enriched regions) and exhausted T cell trajectories. This multimodal collaborative spatial omics data will greatly advance our understanding of the heterogeneity of the immune microenvironment in TDLNs and its dynamic immunomodulatory mechanisms and provide a new direction for targeting and regulating key immune pathways and optimizing immunotherapy strategies.

### 7.3 Clinical translation

LNMPR bridges molecular insights to therapeutic applications. By correlating LN immune maps with treatment responses such as T_pex_ cell activation during neoadjuvant therapy or TDLN remodeling post-radiotherapy, it enables prognostic stratification and personalized intervention. Reversing immunosuppression in metastatic LNs or enhancing antigen-presenting cell function could be prioritized based on LNMPR-guided classification. For example, during the neoadjuvant therapy stage, for patients without LN metastasis, emphasis should be placed on protecting TDLNs to activate systemic immunity. For patients whose LNs have metastasized, it is necessary to consider how to reverse their immunosuppressive state in order to restore their anti-tumor function (111).

Looking ahead, LNMPR establishes a roadmap for LN-centric oncotherapy methodology. Its implementation will require technological innovation (e.g., spatial multi-omics platforms), standardized protocols for LN handling, and clinical trials validating LN-directed therapies. It emphasizes the in-depth understanding of their cellular composition, microenvironment, and cellular interactions through multi-modal analysis, thereby providing a more comprehensive basis for clinical treatment. To mechanistically implement LNMPR, integrated multi-omics data such as spatial omics and single-cell RNA sequencing, are analyzed together in combination using computational tools (e.g., CellPhoneDB, NicheNet) to reconstruct cellular networks within TDLNs. These data can be correlated with clinical outcomes, enabling the prediction of immunotherapy responses and the identification of immunosuppressive regions. Although the LNMPR framework is not widely adopted, emerging studies (e.g., Rahim et al. Cell, 2023) (14) demonstrate its feasibility and provide a paradigm for its application, which provides a paradigm for LNMPR. By integrating single-cell multi-omics and spatial imaging technologies (MIBI), researchers have systematically revealed dynamic changes in CD8^+^ T cell subsets within LNs before and after ICB treatment, as well as their clinical significance. This offers a comprehensive framework that bridges mechanistic insights and practical applications for precision immunotherapy. Emerging strategies, such as modulating Treg activity or targeting endothelial-T cell interactions, holds the potential of reshaping TDLNs microenvironment. Furthermore, the “staged treatment” strategy which preserves non-metastatic LNs to boost systemic immunity while reprogram metastatic LNs may become a new direction for precision oncology. As spatial technologies and immunotherapy evolve, LNMPR will catalyze a paradigm shift toward immune-ecosystem preservation and optimization, ultimately improving patient outcomes in the era of precision medicine.

Although LNMPR holds significant promise, several practical challenges remain for its clinical implementation. Preserving non-metastatic TDLNs demands high surgical precision and real-time assessment to avoid inadvertently removing critical immune hubs. However, current imaging modalities lack sufficient accuracy to detect micro-metastases or accurately evaluate LN immune microenvironments. Potential solutions include intraoperative frozen section analysis, molecular imaging, and rapid multi-parameter detection platforms such as Luminex or Cytometric Bead Array, which can analyze over 100 biomarkers within a few hours and may guide intraoperative decisions. Nonetheless, techniques like ROSE are constrained by the expertise of pathologists, highlighting the need for standardized AI-assisted image analysis. Furthermore, addressing the heterogeneity of metastatic LN responses across cancer types remains a major challenge. While certain tumors present clearly identifiable sentinel lymph nodes that facilitate cross-cancer biomarker discovery, translating these findings into broadly applicable or tumor-specific LN markers will require extensive clinical sample collection and validation. Overcoming these hurdles is essential for fully realizing the potential of LNMPR in precision oncology.

## References

[1] Pereira ER Jones D Jung K Padera TP. The lymph node microenvironment and its role in the progression of metastatic cancer. Semin Cell Dev Biol. (2015) 38:98–105. doi: 10.1016/j.semcdb.2015.01.008, PMID: 25620792 PMC4397158

[2] Halsted WS. I. The results of radical operations for the cure of carcinoma of the breast. Ann Surg. (1907) 46:1–19. doi: 10.1097/00000658-190707000-00001, PMID: 17861990 PMC1414357

[3] Naxerova K Reiter JG Brachtel E Lennerz JK Van De Wetering M Rowan A. Origins of lymphatic and distant metastases in human colorectal cancer. Science. (2017) 357:55–60. doi: 10.1126/science.aai8515, PMID: 28684519 PMC5536201

[4] Venet D Fimereli D Rothé F Boeckx B Maetens M Majjaj S. Phylogenetic reconstruction of breast cancer reveals two routes of metastatic dissemination associated with distinct clinical outcome. EBioMedicine. (2020) 56:102793. doi: 10.1016/j.ebiom.2020.102793, PMID: 32512508 PMC7281848

[5] Giuliano AE Ballman KV McCall L Beitsch PD Brennan MB Kelemen PR. Effect of axillary dissection vs no axillary dissection on 10-year overall survival among women with invasive breast cancer and sentinel node metastasis: the ACOSOG Z0011 (Alliance) randomized clinical trial. JAMA. (2017) 318:918. doi: 10.1001/jama.2017.11470, PMID: 28898379 PMC5672806

[6] Faries MB Thompson JF Cochran AJ Andtbacka RH Mozzillo N Zager JS. Completion dissection or observation for sentinel-node metastasis in melanoma. N Engl J Med. (2017) 376:2211–22. doi: 10.1056/NEJMoa1613210, PMID: 28591523 PMC5548388

[7] Van Pul KM Vuylsteke RJCLM van de Ven R Te Velde EA Rutgers EJT Van Den Tol PM. Selectively hampered activation of lymph node-resident dendritic cells precedes profound T cell suppression and metastatic spread in the breast cancer sentinel lymph node. J Immunother Cancer. (2019) 7:133. doi: 10.1186/s40425-019-0605-1, PMID: 31118093 PMC6530094

[8] Reticker-Flynn NE Engleman EG. Lymph nodes: at the intersection of cancer treatment and progression. Trends Cell Biol. (2023) 33:1021–34. doi: 10.1016/j.tcb.2023.04.001, PMID: 37149414 PMC10624650

[9] Chen DS Mellman I. Oncology meets immunology: the cancer-immunity cycle. Immunity. (2013) 39:1–10. doi: 10.1016/j.immuni.2013.07.012, PMID: 23890059

[10] Prokhnevska N Cardenas MA Valanparambil RM Sobierajska E Barwick BG Jansen C. CD8+ T cell activation in cancer comprises an initial activation phase in lymph nodes followed by effector differentiation within the tumor. Immunity. (2023) 56:107–124.e5. doi: 10.1016/j.immuni.2022.12.002, PMID: 36580918 PMC10266440

[11] Li Y-L Hung W-C. Reprogramming of sentinel lymph node microenvironment during tumor metastasis. J BioMed Sci. (2022) 29:84. doi: 10.1186/s12929-022-00868-1, PMID: 36266717 PMC9583492

[12] Kim R Chang JM Lee H-B Lee SH Kim S-Y Kim ES. Predicting axillary response to neoadjuvant chemotherapy: breast MRI and US in patients with node-positive breast cancer. Radiology. (2019) 293:49–57. doi: 10.1148/radiol.2019190014, PMID: 31407967

[13] Kieran R Goksu M Crocamo S De Paula B. Is it time to retire sentinel lymph node biopsy and use multi-omics prediction models? Ann Transl Med. (2022) 10:655–5. doi: 10.21037/atm-2022-21, PMID: 35845486 PMC9279767

[14] Rahim MK Okholm TLH Jones KB McCarthy EE Liu CC Yee JL. Dynamic CD8+ T cell responses to cancer immunotherapy in human regional lymph nodes are disrupted in metastatic lymph nodes. Cell. (2023) 186:1127–1143.e18. doi: 10.1016/j.cell.2023.02.021, PMID: 36931243 PMC10348701

[15] Fischer JR Jackson HW De Souza N Varga Z Schraml P Moch H. Multiplex imaging of breast cancer lymph node metastases identifies prognostic single-cell populations independent of clinical classifiers. Cell Rep Med. (2023) 4:100977. doi: 10.1016/j.xcrm.2023.100977, PMID: 36921599 PMC10040454

[16] Hiam-Galvez KJ Allen BM Spitzer MH. Systemic immunity in cancer. Nat Rev Cancer. (2021) 21:345–59. doi: 10.1038/s41568-021-00347-z, PMID: 33837297 PMC8034277

[17] Von Renesse J Lin M-C Ho P-C. Tumor-draining lymph nodes – friend or foe during immune checkpoint therapy? Trends Cancer. (2025) S2405803325001049:676–90. doi: 10.1016/j.trecan.2025.04.008, PMID: 40348668

[18] Liu T Liu C Yan M Zhang L Zhang J Xiao M. Single cell profiling of primary and paired metastatic lymph node tumors in breast cancer patients. Nat Commun. (2022) 13:6823. doi: 10.1038/s41467-022-34581-2, PMID: 36357424 PMC9649678

[19] Dong L Hu S Li X Pei S Jin L Zhang L. SPP1^+^ TAM regulates the metastatic colonization of CXCR4^+^ Metastasis-associated tumor cells by remodeling the lymph node microenvironment. Adv Sci. (2024) 11:2400524. doi: 10.1002/advs.202400524, PMID: 39236316 PMC11600252

[20] Lee C Jeong S Jang C Bae H Kim YH Park I. Tumor metastasis to lymph nodes requires YAP-dependent metabolic adaptation. Science. (2019) 363:644–9. doi: 10.1126/science.aav0173, PMID: 30733421

[21] Mao X Tang X Pan H Yu M Ji S Qiu W. B cells and IL-21-producing follicular helper T cells cooperate to determine the dynamic alterations of premetastatic tumor draining lymph nodes of breast cancer. Res Wash DC. (2024) 7:346. doi: 10.34133/research.0346, PMID: 38559676 PMC10981934

[22] Xin S Liu X Li Z Sun X Wang R Zhang Z. ScRNA-seq revealed an immunosuppression state and tumor microenvironment heterogeneity related to lymph node metastasis in prostate cancer. Exp Hematol Oncol. (2023) 12:49. doi: 10.1186/s40164-023-00407-0, PMID: 37221625 PMC10204220

[23] Lao S Chen Z Wang W Zheng Y Xiong S He P. Prognostic patterns in invasion lymph nodes of lung adenocarcinoma reveal distinct tumor microenvironments. NPJ Precis Oncol. (2024) 8:164. doi: 10.1038/s41698-024-00639-1, PMID: 39080406 PMC11289302

[24] Cabrita R Lauss M Sanna A Donia M Skaarup Larsen M Mitra S. Tertiary lymphoid structures improve immunotherapy and survival in melanoma. Nature. (2020) 577:561–5. doi: 10.1038/s41586-019-1914-8, PMID: 31942071

[25] Riedel A Shorthouse D Haas L Hall BA Shields J. Tumor-induced stromal reprogramming drives lymph node transformation. Nat Immunol. (2016) 17:1118–27. doi: 10.1038/ni.3492, PMID: 27400148 PMC4994871

[26] Das S Sarrou E Podgrabinska S Cassella M Mungamuri SK Feirt N. Tumor cell entry into the lymph node is controlled by CCL1 chemokine expressed by lymph node lymphatic sinuses. J Exp Med. (2013) 210:1509–28. doi: 10.1084/jem.20111627, PMID: 23878309 PMC3727324

[27] Rossi M Altea-Manzano P Demicco M Doglioni G Bornes L Fukano M. PHGDH heterogeneity potentiates cancer cell dissemination and metastasis. Nature. (2022) 605:747–53. doi: 10.1038/s41586-022-04758-2, PMID: 35585241 PMC9888363

[28] Gu Y Liu Y Fu L Zhai L Zhu J Han Y. Tumor-educated B cells selectively promote breast cancer lymph node metastasis by HSPA4-targeting IgG. Nat Med. (2019) 25:312–22. doi: 10.1038/s41591-018-0309-y, PMID: 30643287

[29] Pardoll DM. The blockade of immune checkpoints in cancer immunotherapy. Nat Rev Cancer. (2012) 12:252–64. doi: 10.1038/nrc3239, PMID: 22437870 PMC4856023

[30] Shindo Y Yoshimura K Kuramasu A Watanabe Y Ito H Kondo T. Combination immunotherapy with 4-1BB activation and PD-1 blockade enhances antitumor efficacy in a mouse model of subcutaneous tumor. Anticancer Res. (2015) 35:129–36. doi: 10.1038/nrc3239, PMID: 25550543

[31] Chen L Flies DB. Molecular mechanisms of T cell co-stimulation and co-inhibition. Nat Rev Immunol. (2013) 13:227–42. doi: 10.1038/nri3405, PMID: 23470321 PMC3786574

[32] Chen L Flies DB. Molecular mechanisms of T cell co-stimulation and co-inhibition. Nat Rev Immunol. (2013) 13:227–42. doi: 10.1038/nri3405, PMID: 23470321 PMC3786574

[33] Saxena V Li L Paluskievicz C Kasinath V Bean A Abdi R. Role of lymph node stroma and microenvironment in T cell tolerance. Immunol Rev. (2019) 292:9–23. doi: 10.1111/imr.12799, PMID: 31538349 PMC6935411

[34] Ji M Liu Y Li Q Li X-D Zhao W-Q Zhang H. PD-1/PD-L1 pathway in non-small-cell lung cancer and its relation with EGFR mutation. J Transl Med. (2015) 13:5. doi: 10.1186/s12967-014-0373-0, PMID: 25592115 PMC4302082

[35] Keir ME Butte MJ Freeman GJ Sharpe AH. PD-1 and its ligands in tolerance and immunity. Annu Rev Immunol. (2008) 26:677–704. doi: 10.1146/annurev.immunol.26.021607.090331, PMID: 18173375 PMC10637733

[36] Zhang Y Kang S Shen J He J Jiang L Wang W. Prognostic significance of programmed cell death 1 (PD-1) or PD-1 ligand 1 (PD-L1) Expression in epithelial-originated cancer: a meta-analysis. Med (Baltimore). (2015) 94:e515. doi: 10.1097/MD.0000000000000515, PMID: 25674748 PMC4602735

[37] Agata Y Kawasaki A Nishimura H Ishida Y Tsubata T Yagita H. Expression of the PD-1 antigen on the surface of stimulated mouse T and B lymphocytes. Int Immunol. (1996) 8:765–72. doi: 10.1093/intimm/8.5.765, PMID: 8671665

[38] Ritprajak P Azuma M. Intrinsic and extrinsic control of expression of the immunoregulatory molecule PD-L1 in epithelial cells and squamous cell carcinoma. Oral Oncol. (2015) 51:221–8. doi: 10.1016/j.oraloncology.2014.11.014, PMID: 25500094

[39] He X Xu C. Immune checkpoint signaling and cancer immunotherapy. Cell Res. 30(8):660–9. Available online at: https://www.nature.com/articles/s41422-020-0343-4. 10.1038/s41422-020-0343-4 PMC7395714 32467592

[40] Robert C Schachter J Long GV Arance A Grob JJ Mortier L. Pembrolizumab versus ipilimumab in advanced melanoma. N Engl J Med. (2015) 372:2521–32. doi: 10.1056/NEJMoa1503093, PMID: 25891173

[41] Topalian SL Sznol M McDermott DF Kluger HM Carvajal RD Sharfman WH. Survival, durable tumor remission, and long-term safety in patients with advanced melanoma receiving nivolumab. J Clin Oncol Off J Am Soc Clin Oncol. (2014) 32:1020–30. doi: 10.1200/JCO.2013.53.0105, PMID: 24590637 PMC4811023

[42] Reck M Remon J Hellmann MD. First-line immunotherapy for non-small-cell lung cancer. J Clin Oncol Off J Am Soc Clin Oncol. (2022) 40:586–97. doi: 10.1200/JCO.21.01497, PMID: 34985920

[43] Howlader N Forjaz G Mooradian MJ Meza R Kong CY Cronin KA. The effect of advances in lung-cancer treatment on population mortality. N Engl J Med. (2020) 383:640–9. doi: 10.1056/NEJMoa1916623, PMID: 32786189 PMC8577315

[44] Motzer RJ Rini BI McDermott DF Redman BG Kuzel TM Harrison MR. Nivolumab for metastatic renal cell carcinoma: results of a randomized phase II trial. J Clin Oncol Off J Am Soc Clin Oncol. (2015) 33:1430–7. doi: 10.1200/JCO.2014.59.0703, PMID: 25452452 PMC4806782

[45] Robert C Long GV Brady B Dutriaux C Maio M Mortier L. Nivolumab in previously untreated melanoma without BRAF mutation. N Engl J Med. (2015) 372:320–30. doi: 10.1056/NEJMoa1412082, PMID: 25399552

[46] Vasudev NS Ainsworth G Brown S Pickering L Waddell T Fife K. Standard versus modified ipilimumab, in combination with nivolumab, in advanced renal cell carcinoma: A randomized phase II trial (PRISM). J Clin Oncol. (2024) 42(3):312–23. doi: 10.1200/JCO.23.00236, PMID: 37931206 PMC10824383

[47] Liu Y Hu Y Xue J Li J Yi J Bu J. Advances in immunotherapy for triple-negative breast cancer. Mol Cancer. (2023) 22:145. doi: 10.1186/s12943-023-01850-7, PMID: 37660039 PMC10474743

[48] Adams S Schmid P Rugo HS Winer EP Loirat D Awada A. Pembrolizumab monotherapy for previously treated metastatic triple-negative breast cancer: cohort A of the phase II KEYNOTE-086 study. Ann Oncol Off J Eur Soc Med Oncol. (2019) 30:397–404. doi: 10.1093/annonc/mdy517, PMID: 30475950

[49] Roberts EW Broz ML Binnewies M Headley MB Nelson AE Wolf DM. Critical role for CD103(+)/CD141(+) dendritic cells bearing CCR7 for tumor antigen trafficking and priming of T cell immunity in melanoma. Cancer Cell. (2016) 30:324–36. doi: 10.1016/j.ccell.2016.06.003, PMID: 27424807 PMC5374862

[50] Chuah S Lee J Song Y Kim H-D Wasser M Kaya NA. Uncoupling immune trajectories of response and adverse events from anti-PD-1 immunotherapy in hepatocellular carcinoma. J Hepatol. (2022) 77:683–94. doi: 10.1016/j.jhep.2022.03.039, PMID: 35430299

[51] Garris CS Arlauckas SP Kohler RH Trefny MP Garren S Piot C. Successful anti-PD-1 cancer immunotherapy requires T cell-dendritic cell crosstalk involving the cytokines IFN-γ and IL-12. Immunity. (2018) 49:1148–1161.e7. doi: 10.1016/j.immuni.2018.09.024, PMID: 30552023 PMC6301092

[52] Meiser P Knolle MA Hirschberger A De Almeida GP Bayerl F Lacher S. A distinct stimulatory cDC1 subpopulation amplifies CD8+ T cell responses in tumors for protective anti-cancer immunity. Cancer Cell. (2023) 41:1498–1515.e10. doi: 10.1016/j.ccell.2023.06.008, PMID: 37451271

[53] Duong E Fessenden TB Lutz E Dinter T Yim L Blatt S. Type I interferon activates MHC class I-dressed CD11b+ conventional dendritic cells to promote protective anti-tumor CD8+ T cell immunity. Immunity. (2022) 55:308–323.e9. doi: 10.1016/j.immuni.2021.10.020, PMID: 34800368 PMC10827482

[54] Asano K Nabeyama A Miyake Y Qiu C-H Kurita A Tomura M. CD169-positive macrophages dominate antitumor immunity by crosspresenting dead cell-associated antigens. Immunity. (2011) 34:85–95. doi: 10.1016/j.immuni.2010.12.011, PMID: 21194983

[55] Wang L Guo W Guo Z Yu J Tan J Simons DL. PD-L1-expressing tumor-associated macrophages are immunostimulatory and associate with good clinical outcome in human breast cancer. Cell Rep Med. (2024) 5:101420. doi: 10.1016/j.xcrm.2024.101420, PMID: 38382468 PMC10897617

[56] Lin X Kang K Chen P Zeng Z Li G Xiong W. Regulatory mechanisms of PD-1/PD-L1 in cancers. Mol Cancer. (2024) 23:108. doi: 10.1186/s12943-024-02023-w, PMID: 38762484 PMC11102195

[57] Mellman I Chen DS Powles T Turley SJ. The cancer-immunity cycle: Indication, genotype, and immunotype. Immunity. (2023) 56:2188–205. doi: 10.1016/j.immuni.2023.09.011, PMID: 37820582

[58] Connolly KA Kuchroo M Venkat A Khatun A Wang J William I. A reservoir of stem-like CD8+ T cells in the tumor-draining lymph node preserves the ongoing antitumor immune response. Sci Immunol. (2021) 6:eabg7836. doi: 10.1126/sciimmunol.abg7836, PMID: 34597124 PMC8593910

[59] Huang Q Wu X Wang Z Chen X Wang L Lu Y. The primordial differentiation of tumor-specific memory CD8+ T cells as bona fide responders to PD-1/PD-L1 blockade in draining lymph nodes. Cell. (2022) 185:4049–4066.e25. doi: 10.1016/j.cell.2022.09.020, PMID: 36208623

[60] Speiser DE Chijioke O Schaeuble K Münz C. CD4+ T cells in cancer. Nat Cancer. (2023) 4:317–29. doi: 10.1038/s43018-023-00521-2, PMID: 36894637

[61] Ferris ST Durai V Wu R Theisen DJ Ward JP Bern MD. cDC1 prime and are licensed by CD4+ T cells to induce anti-tumour immunity. Nature. (2020) 584:624–9. doi: 10.1038/s41586-020-2611-3, PMID: 32788723 PMC7469755

[62] Montauti E Oh DY Fong L. CD4+ T cells in antitumor immunity. Trends Cancer. (2024) 10:969–85. doi: 10.1016/j.trecan.2024.07.009, PMID: 39242276 PMC11464182

[63] Mintz MA Cyster JG. T follicular helper cells in germinal center B cell selection and lymphomagenesis. Immunol Rev. (2020) 296:48–61. doi: 10.1111/imr.12860, PMID: 32412663 PMC7817257

[64] Delclaux I Ventre KS Jones D Lund AW. The tumor-draining lymph node as a reservoir for systemic immune surveillance. Trends Cancer. (2024) 10:28–37. doi: 10.1016/j.trecan.2023.09.006, PMID: 37863720 PMC10843049

[65] Núñez NG Tosello Boari J Ramos RN Richer W Cagnard N Anderfuhren CD. Tumor invasion in draining lymph nodes is associated with Treg accumulation in breast cancer patients. Nat Commun. (2020) 11:3272. doi: 10.1038/s41467-020-17046-2, PMID: 32601304 PMC7324591

[66] Francis DM Manspeaker MP Schudel A Sestito LF O’Melia MJ Kissick HT. Blockade of immune checkpoints in lymph nodes through locoregional delivery augments cancer immunotherapy. Sci Transl Med. (2020) 12:eaay3575. doi: 10.1126/scitranslmed.aay3575, PMID: 32998971 PMC8377700

[67] Reticker-Flynn NE Zhang W Belk JA Basto PA Escalante NK Pilarowski GOW. Lymph node colonization induces tumor-immune tolerance to promote distant metastasis. Cell. (2022) 185:1924–1942.e23. doi: 10.1016/j.cell.2022.04.019, PMID: 35525247 PMC9149144

[68] Hollern DP Xu N Thennavan A Glodowski C Garcia-Recio S Mott KR. B cells and T follicular helper cells mediate response to checkpoint inhibitors in high mutation burden mouse models of breast cancer. Cell. (2019) 179:1191–1206.e21. doi: 10.1016/j.cell.2019.10.028, PMID: 31730857 PMC6911685

[69] Niu L Liu Z Liu G Li M Zong X Wang D. Surface hydrophobic modification enhanced catalytic performance of electrochemical nitrogen reduction reaction. Nano Res. (2022) 15:3886–93. doi: 10.1007/s12274-021-4015-6

[70] Bod L Kye Y-C Shi J Torlai Triglia E Schnell A Fessler J. B-cell-specific checkpoint molecules that regulate anti-tumour immunity. Nature. (2023) 619:348–56. doi: 10.1038/s41586-023-06231-0, PMID: 37344597 PMC10795478

[71] He M Roussak K Ma F Borcherding N Garin V White M. CD5 expression by dendritic cells directs T cell immunity and sustains immunotherapy responses. Science. (2023) 379:eabg2752. doi: 10.1126/science.abg2752, PMID: 36795805 PMC10424698

[72] Sade-Feldman M Yizhak K Bjorgaard SL Ray JP De Boer CG Jenkins RW. Defining T cell states associated with response to checkpoint immunotherapy in melanoma. Cell. (2018) 175:998–1013.e20. doi: 10.1016/j.cell.2018.10.038, PMID: 30388456 PMC6641984

[73] Molodtsov AK Khatwani N Vella JL Lewis KA Zhao Y Han J. Resident memory CD8+ T cells in regional lymph nodes mediate immunity to metastatic melanoma. Immunity. (2021) 54:2117–2132.e7. doi: 10.1016/j.immuni.2021.08.019, PMID: 34525340 PMC9015193

[74] Helmink BA Reddy SM Gao J Zhang S Basar R Thakur R. B cells and tertiary lymphoid structures promote immunotherapy response. Nature. (2020) 577:549–55. doi: 10.1038/s41586-019-1922-8, PMID: 31942075 PMC8762581

[75] Maier B Leader AM Chen ST Tung N Chang C LeBerichel J. A conserved dendritic-cell regulatory program limits antitumour immunity. Nature. (2020) 580:257–62. doi: 10.1038/s41586-020-2134-y, PMID: 32269339 PMC7787191

[76] Jiang W Chan CK Weissman IL Kim BYS Hahn SM. Immune priming of the tumor microenvironment by radiation. Trends Cancer. (2016) 2:638–45. doi: 10.1016/j.trecan.2016.09.007, PMID: 28741502

[77] Lugade AA Sorensen EW Gerber SA Moran JP Frelinger JG Lord EM. Radiation-induced IFN-γ Production within the tumor microenvironment influences antitumor immunity1. J Immunol. (2008) 180:3132–9. doi: 10.4049/jimmunol.180.5.3132, PMID: 18292536

[78] Zhang S-N Choi I-K Huang J-H Yoo J-Y Choi K-J Yun C-O. Optimizing DC vaccination by combination with oncolytic adenovirus coexpressing IL-12 and GM-CSF. Mol Ther. (2011) 19:1558–68. doi: 10.1038/mt.2011.29, PMID: 21468000 PMC3149171

[79] Thakur BK Zhang H Becker A Matei I Huang Y Costa-Silva B. Double-stranded DNA in exosomes: a novel biomarker in cancer detection. Cell Res. (2014) 24:766–9. doi: 10.1038/cr.2014.44, PMID: 24710597 PMC4042169

[80] Khalifa J Thébault N Scarlata C-M Norkowski E Massabeau C Brouchet L. Immune changes in hilar tumor draining lymph nodes following node sparing neoadjuvant chemoradiotherapy of localized cN0 non-small cell lung cancer. Front Oncol. (2023) 13:1269166. doi: 10.3389/fonc.2023.1269166, PMID: 38074683 PMC10699862

[81] Denis F Garaud P Bardet E Alfonsi M Sire C Germain T. Final results of the 94–01 french head and neck oncology and radiotherapy group randomized trial comparing radiotherapy alone with concomitant radiochemotherapy in advanced-stage oropharynx carcinoma. J Clin Oncol. (2004) 22:69–76. doi: 10.1200/JCO.2004.08.021, PMID: 14657228

[82] Bernier J Domenge C Ozsahin M Matuszewska K Lefèbvre J-L Greiner RH. Postoperative irradiation with or without concomitant chemotherapy for locally advanced head and neck cancer. N Engl J Med. (2004) 350:1945–52. doi: 10.1056/NEJMoa032641, PMID: 15128894

[83] Weichselbaum RR Liang H Deng L Fu Y-X. Radiotherapy and immunotherapy: a beneficial liaison? Nat Rev Clin Oncol. (2017) 14:365–79. doi: 10.1038/nrclinonc.2016.211, PMID: 28094262

[84] Su J Li S Zhou X Zhang Z Yan Y Liu S. Chemotherapy-induced metastasis: molecular mechanisms and clinical therapies. Acta Pharmacol Sin. (2023) 44:1725–36. doi: 10.1038/s41401-023-01093-8, PMID: 37169853 PMC10462662

[85] Anand U Dey A Chandel AKS Sanyal R Mishra A Pandey DK. Cancer chemotherapy and beyond: Current status, drug candidates, associated risks and progress in targeted therapeutics. Genes Dis. (2023) 10:1367–401. doi: 10.1016/j.gendis.2022.02.007, PMID: 37397557 PMC10310991

[86] Tacar O Sriamornsak P Dass CR. Doxorubicin: an update on anticancer molecular action, toxicity and novel drug delivery systems. J Pharm Pharmacol. (2013) 65:157–70. doi: 10.1111/j.2042-7158.2012.01567.x, PMID: 23278683

[87] Weaver BA. How Taxol/paclitaxel kills cancer cells. Mol Biol Cell. (2014) 25:2677–81. doi: 10.1091/mbc.e14-04-0916, PMID: 25213191 PMC4161504

[88] Du Bois H Heim TA Lund AW. Tumor-draining lymph nodes: At the crossroads of metastasis and immunity. Sci Immunol. (2021) 6:eabg3551. doi: 10.1126/sciimmunol.abg3551, PMID: 34516744 PMC8628268

[89] Nishino M Ramaiya NH Hatabu H Hodi FS. Monitoring immune-checkpoint blockade: response evaluation and biomarker development. Nat Rev Clin Oncol. (2017) 14:655–68. doi: 10.1038/nrclinonc.2017.88, PMID: 28653677 PMC5650537

[90] Kroemer G Galluzzi L Kepp O Zitvogel L. Immunogenic cell death in cancer therapy. Annu Rev Immunol. (2013) 31:51–72. doi: 10.1146/annurev-immunol-032712-100008, PMID: 23157435

[91] Yin G Liu L Yu T Yu L Feng M Zhou C. Genomic and transcriptomic analysis of breast cancer identifies novel signatures associated with response to neoadjuvant chemotherapy. Genome Med. (2024) 16:11. doi: 10.1186/s13073-024-01286-8, PMID: 38217005 PMC10787499

[92] Wang Z Till B Gao Q. Chemotherapeutic agent-mediated elimination of myeloid-derived suppressor cells. Oncoimmunology. (2017) 6:e1331807. doi: 10.1080/2162402X.2017.1331807, PMID: 28811975 PMC5543863

[93] Verma R Foster RE Horgan K Mounsey K Nixon H Smalle N. Lymphocyte depletion and repopulation after chemotherapy for primary breast cancer. Breast Cancer Res. (2016) 18:10. doi: 10.1186/s13058-015-0669-x, PMID: 26810608 PMC4727393

[94] Chen X Ling X Xia J Zhu Y Zhang L He Y. Mature dendritic cell-derived dendrosomes swallow oxaliplatin-loaded nanoparticles to boost immunogenic chemotherapy and tumor antigen-specific immunotherapy. Bioact Mater. (2022) 15:15–28. doi: 10.1016/j.bioactmat.2021.12.020, PMID: 35386340 PMC8941172

[95] Garg AD Agostinis P. Cell death and immunity in cancer: From danger signals to mimicry of pathogen defense responses. Immunol Rev. (2017) 280:126–48. doi: 10.1111/imr.12574, PMID: 29027218

[96] Zhang L Dermawan K Jin M Liu R Zheng H Xu L. Differential impairment of regulatory T cells rather than effector T cells by paclitaxel-based chemotherapy. Clin Immunol. (2008) 129:219–29. doi: 10.1016/j.clim.2008.07.013, PMID: 18771959

[97] Emens LA Middleton G. The interplay of immunotherapy and chemotherapy: harnessing potential synergies. Cancer Immunol Res. (2015) 3:436–43. doi: 10.1158/2326-6066.CIR-15-0064, PMID: 25941355 PMC5012642

[98] Schmid P Adams S Rugo HS Schneeweiss A Barrios CH Iwata H. Atezolizumab and nab-paclitaxel in advanced triple-negative breast cancer. N Engl J Med. (2018) 379:2108–21. doi: 10.1056/NEJMoa1809615, PMID: 30345906

[99] Loibl S O’Shaughnessy J Untch M Sikov WM Rugo HS McKee MD. Addition of the PARP inhibitor veliparib plus carboplatin or carboplatin alone to standard neoadjuvant chemotherapy in triple-negative breast cancer (BrighTNess): a randomised, phase 3 trial. Lancet Oncol. (2018) 19:497–509. doi: 10.1016/S1470-2045(18)30111-6, PMID: 29501363

[100] Gianni L Eiermann W Semiglazov V Manikhas A Lluch A Tjulandin S. Neoadjuvant chemotherapy with trastuzumab followed by adjuvant trastuzumab versus neoadjuvant chemotherapy alone, in patients with HER2-positive locally advanced breast cancer (the NOAH trial): a randomised controlled superiority trial with a parallel HER2-negative cohort. Lancet. (2010) 375:377–84. doi: 10.1016/S0140-6736(09)61964-4, PMID: 20113825

[101] Zitvogel L Apetoh L Ghiringhelli F Kroemer G. Immunological aspects of cancer chemotherapy. Nat Rev Immunol. (2008) 8:59–73. doi: 10.1038/nri2216, PMID: 18097448

[102] Blenman KRM He T-F Frankel PH Ruel NH Schwartz EJ Krag DN. Sentinel lymph node B cells can predict disease-free survival in breast cancer patients. NPJ Breast Cancer. (2018) 4:28. doi: 10.1038/s41523-018-0081-7, PMID: 30155518 PMC6107630

[103] Chen B Zhang G Lai J Xiao W Li X Li C. Genetic and immune characteristics of sentinel lymph node metastases and multiple lymph node metastases compared to their matched primary breast tumours. eBioMedicine. (2021) 71:103542. doi: 10.1016/j.ebiom.2021.103542, PMID: 34454403 PMC8399410

[104] Carmeliet P Jain RK. Principles and mechanisms of vessel normalization for cancer and other angiogenic diseases. Nat Rev Drug Discov. (2011) 10:417–27. doi: 10.1038/nrd3455, PMID: 21629292

[105] Verma S Miles D Gianni L Krop IE Welslau M Baselga J. Trastuzumab emtansine for HER2-positive advanced breast cancer. N Engl J Med. (2012) 367:1783–91. doi: 10.1056/NEJMoa1209124, PMID: 23020162 PMC5125250

[106] Dammeijer F Van Gulijk M Mulder EE Lukkes M Klaase L Van Den Bosch T. The PD-1/PD-L1-checkpoint restrains T cell immunity in tumor-draining lymph nodes. Cancer Cell. (2020) 38:685–700.e8. doi: 10.1016/j.ccell.2020.09.001, PMID: 33007259

[107] Goode EF Roussos Torres ET Irshad S. Lymph node immune profiles as predictive biomarkers for immune checkpoint inhibitor response. Front Mol Biosci. (2021) 8:674558. doi: 10.3389/fmolb.2021.674558, PMID: 34141724 PMC8205515

[108] Zhang Y Deng C Zheng Q Qian B Ma J Zhang C. Selective mediastinal lymph node dissection strategy for clinical T1N0 invasive lung cancer: A prospective, multicenter, clinical trial. J Thorac Oncol. (2023) 18:931–9. doi: 10.1016/j.jtho.2023.02.010, PMID: 36841542

[109] Earland N Semenkovich NP Ramirez RJ Gerndt SP Harris PK Gu Z. Sensitive MRD detection from lymphatic fluid after surgery in HPV-associated oropharyngeal cancer. Clin Cancer Res. (2024) 30:1409–21. doi: 10.1158/1078-0432.CCR-23-1789, PMID: 37939112 PMC10982646

[110] Guo P Mao L Chen Y Lee CN Cardilla A Li M. Multiplexed spatial mapping of chromatin features, transcriptome and proteins in tissues. Nat Methods. (2025) 22:520–9. doi: 10.1038/s41592-024-02576-0, PMID: 39870864 PMC11906265

[111] Fransen MF Schoonderwoerd M Knopf P Camps MGM Hawinkels LJAC Kneilling M. Tumor-draining lymph nodes are pivotal in PD-1/PD-L1 checkpoint therapy. JCI Insight. (2018) 3:e124507. doi: 10.1172/jci.insight.124507, PMID: 30518694 PMC6328025

